# Efficacy and safety of colonic dialysis combined with traditional Chinese medicine retention enema in the treatment of chronic renal failure: A protocol for systematic review and meta-analysis

**DOI:** 10.1097/MD.0000000000028082

**Published:** 2021-12-17

**Authors:** Zhilei Wang, Shoulin Zhang, Xue Zheng, Lili Zhang

**Affiliations:** a Department of Traditional Chinese Medicine, Changchun University of Chinese Medicine, Changchun, Jilin Province, China; b Department of Nephrology, Jilin Province Hospital of Chinese Medicine: First Affiliated Hospital to Changchun University of Chinese Medicine, Changchun, Jilin Province, China.

**Keywords:** chronic renal failure, colonic dialysis, meta-analysis, protocol, systematic review, traditional Chinese medicinal enemas

## Abstract

**Background:**

Chronic renal failure (CRF) is a major public health problem worldwide nowadays. It is characterized by a slow reduction in kidney function identified by an increase of serum creatinine levels and a reduction of urine output. CRF is easier to diagnose than to treat. Clinical evidence shows that colonic dialysis combined with traditional Chinese medicine (TCM) enema can treat CRF by reducing serum creatinine. To assess the therapeutic efficacy and safety of colonic dialysis combined with Traditional Chinese medicine retention enema in CRF, we created a protocol for a systematic review to inform future clinical applications.

**Methods:**

Eligible random controlled trials were collected from 8 bibliographic databases (PubMed, EMBASE, Web of Science, The Cochrane Library, Chinese Biomedical Literature Database, Chinese National Knowledge Infrastructure, Chinese Science and Technology Periodical Database, Wanfang Database), completed before October 2021. The primary outcome is the serum creatinine, Urea nitrogen, total effective rate, uric acid, creatinine clearance. Secondary outcome: TCM syndrome score, glomerular filtration rate, hemoglobin, adverse reactions, and adverse events. Data extraction and quality assessment were independently conducted by 2 researchers. The quality and bias of the data were assessed using RevMan5.4 software.

**Results:**

This study will provide a clinical basis for colonic dialysis combined with TCM retention enema in the treatment of CRF.

**Conclusion:**

Colonic dialysis combined with TCM retention enema in the treatment of CRF can delay the progression of renal disease, proving its effectiveness and safety.

**INPLASY Registration number:**

INPLASY2021100116

## 1 Introduction

Nowadays, chronic renal failure (CRF) is a significant public health problem worldwide.^[1]^ It is not an independent disease but is caused by various causes of kidney damage and progressive deterioration. It is characterized by a decrease in renal function that leads to irreversible nephrosclerosis and nephron loss.^[2]^ When it reaches the end stage, that renal function accounts for only 10% to 15% of normal, a series of clinical symptoms appear. Lesions often present progress, gradually developing to end-stage renal disease after renal replacement therapy, which makes a tremendous threat to human health, and seriously affects the quality of human survival, and cause more burden to the society and family. With the development of society, human lifestyles are changed; unfortunately, the incidence of CRF is increasing. According to statistics, the prevalence of CRF in American adults is highly 15.1%, and the prevalence of end-stage renal disease is 1738 per million population. In China, some reports show that the prevalence rate of CRF is about 10.8%, and the incidence rate of male and female is 55% and 45% respectively.^[3]^ For Western medicine, the treatments only focus on the basic diseases, preventing complications and giving invasive kidney replacement therapy. Subsequently, its therapeutic effect is limited. How to delay the further development of kidney disease as far as possible is an urgent and difficult problem. Uremia period has to undergo renal replacement therapy, but dialysis needs highly content complications, and the cost is expensive. Therefore, how to effectively reduce serum toxin level, delay the process of CRF, and improve the quality of patients’ life of patients are meeting great challenges.

Referring to domestic and foreign literature,^[4–7]^ modern nephropathy-related scholars have done a number of studies on the relationship between the intestinal tract and kidney. They put forward the theory of gut-kidney axis to explain the mechanism of CRF. These studies indicated that intestinal microbiota is the main source of uremia toxin, and changes in intestinal microbiota can lead to a series of abnormal changes in nephropathy and various complications. Also, it was emphasized that intestinal microbiota is the central link of the enteric-renal axis.

Under normal circumstances, the metabolites and toxins of human body are mainly excreted through urine, intestinal, skin, and respiration, among which urine and intestinal tract undertake the majority. Urea nitrogen is the main end product of protein metabolism, creatinine is the product of muscle metabolism, and uric acid is the final product of purine degradation. These 3 substances are mainly discharged from urine through the kidney.

However, when CRF occurs, the detoxification function of the intestinal tract will also increase compensatory to assist the body to eliminate toxins. Therefore, intestinal do assistance to detoxification therapy for CRF and then can effectively delay the progression of CRF.

Colonic dialysis combined with traditional Chinese medicine (TCM) retention enema is green therapy. They do not need oral administration, and they have less harm, satisfactory affections, and are easy to be accepted by patients. Colon dialysis: colonic mucosa as a semipermeable membrane, to infuse dialysate and lumen and use within the colon mucosa capillary blood inside with dialysate concentration and osmotic pressure gradient difference, through the principle of diffusion and permeation removes toxins and necessary supplement material.^[8]^ Chinese medicine retention enema improves the renal function of CRF. Collectively, CRF is a long course and difficult disease with various clinical manifestations and irreversible renal injury, etc. The advantage lies in that the prescription of TCM treatment of CRF is flexible, the dosage form is diverse, and side effects are less. Therefore, promoting a variety of green TCM therapy in combination with the treatment of CRF and improving patient quality of life, are the clinical inevitable trend. However, there is no systematic review and analysis of existing clinical data to determine the safety and effectiveness of colonic dialysis-TCM retention enema combined therapy.^[9,10]^ Therefore, the proposal of this study can solve this problem. We conducted a systematic review and meta-analysis of published randomized clinical trials of colonic dialysis-TCM retention enema to gather scientific evidence on the safety and efficacy of the treatment and to obtain a reference protocol to guide its clinical use.

### 1.1 Protocol registration

This study will be reported following the Preferred Reporting Project Statement Guidelines for Systematic Reviews and Meta-Analysis Agreements.^[11,12]^ The protocol for this systematic review has been registered on INPLASY. website (https://inplasy.com/inplasy-2021-10-0116/), the registration number: INPLASY2021100116).

### 1.2 Inclusion criteria

#### 1.2.1 Study design

All randomized controlled trials using colonic dialysis combined with traditional Chinese medicine retention enema to treat chronic renal failure will be accepted. No language or publication status requirements. In addition, relevant nonrandomized controls, reviews, individual cases, etc, were excluded.

#### 1.2.2 Types of patients

Patients diagnosed with chronic renal failure will be included, regardless of race, gender, age.

#### 1.2.3 Interventions and comparisons

The patients in the control group received routine treatment for chronic renal disease, above which the treatment group used colon dialysis combined with Chinese medicine retention enema.

#### 1.2.4 Outcomes

##### 1.2.4.1 Primary outcomes

- Serum creatinine.
- Urea nitrogen.
- Total effective rate.
- Uric acid.
- Creatinine clearance.

##### 1.2.4.2 Secondary outcomes

- TCM syndrome score.
- Glomerular filtration rate.
- Hemoglobin.
- Adverse reactions and adverse events.

### 1.3 Exclusion criteria

- Does not meet diagnostic criteria or has no diagnostic criteria.
- No according to the group or the test design is flawed, or the statistical method is improper.
- Not available literature on outcome indicators.
- Animal experiment, review, experience summary, case report.
- Case review and retrospective study.
- Studies without full text or lacking original data.

### 1.4 Database search strategy

We conducted a systematic search for relevant documents in the Chinese and English databases, and the search time is limited to October 31, 2021. The following 8 databases are included: PubMed, EMBASE, Web of Science, The Cochrane Library, Chinese Biomedical Literature Database, Chinese National Knowledge Infrastructure, Chinese Science, and Technology Periodical Database, Wanfang Database. Relevant journals were searched to trace the references included in the study. We will be using the following keywords “chronic renal failure,” “meta-analysis,” “colonic dialysis,” “traditional Chinese medicinal enemas.” We performed a search for ongoing unpublished trials in INPLASY.COM. In addition, other resources will be searched if necessary.

### 1.5 Study selection

**Figure 1:**
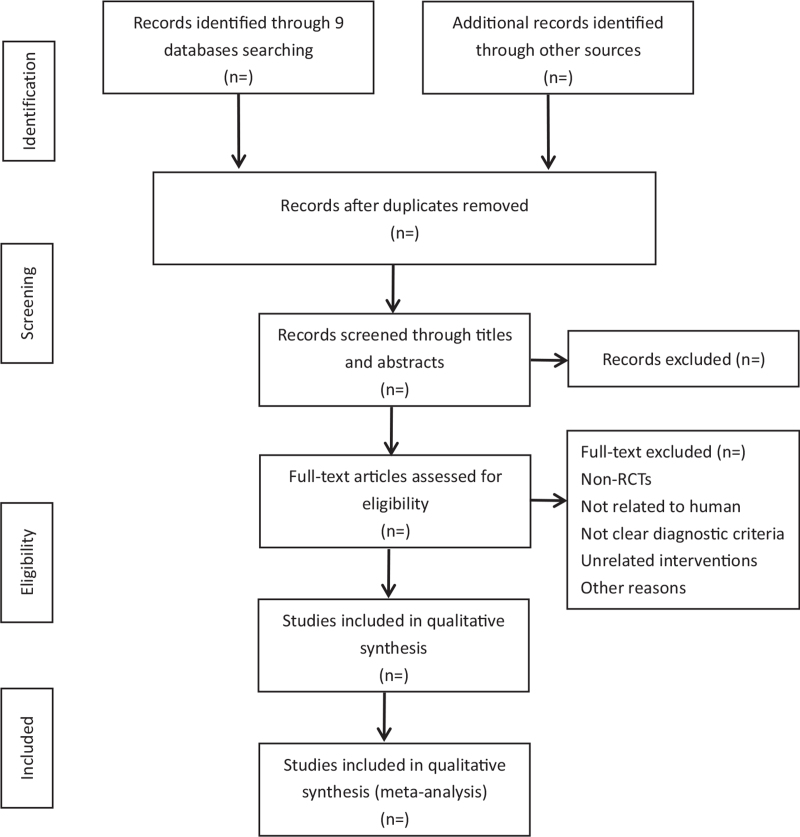
Flow diagram of study selection process.

Endnote X9 was used for the electronic management of the literature. Following the removal of duplicates, pairs of reviewers independently screened titles and abstracts and reviewed the full texts to identify qualified studies. Where there was a lack of consensus between the 2 reviewers, any disagreements were resolved through discussion with a third reviewer (Fig. 1 the screening process).

### 1.6 Assessment of study quality

Two searchers searched the literature independently and checked one by one included in the study. If there is disagreement about the inclusion of the study, consult the expert to decide which one should be used. Try to avoid missed detection. Cochrane systems reviewers have used the Volume 5.1 Bias risk Assessment tool. The included literature was qualified from the following 6 aspects of quantitative evaluation. Whether random sequence generation is sufficient; whether the allocation is hidden; whether the blind method is used; whether the result data is complete; selective reporting; whether there are publication bias.

### 1.7 Statistical analysis

Review Manager software (v5.4; Cochrane Collaboration) is used for the meta-analysis. Meta-analysis is a combination of multiple independent studies, and only homogeneous data can be combined statistically. Therefore, the Chi-square test is used to detect the heterogeneity of the tests. If the heterogeneity test result is homogeneity (*P* > .05), the fixed-effects model is used. For heterogeneity (*P* < .05), random effects model was used. Odds ratio and 95% confidence interval were calculated from counting data. Weighted mean difference and 95% confidence interval were selected when the measurement data were in the same unit of weights and measures, while standardized mean difference (SMD) and its 95% confidence interval are used when different units of measurement are used for outcome measurements.

### 1.8 Subgroup analysis

All the information is collected from the study, potential heterogeneity is inevitable, to reduce the impact of the results, we could conduct subgroup analysis according to the sex, age, and treatment duration of all included subjects.

### 1.9 Sensitivity analysis

It is necessary to conduct sensitivity analysis, avoid the results being affected by high-risk bias, delete low-quality results, and improve the reliability of the data.

### 1.10 Ethics and dissemination

Due to the study, data will get from public databases; it does not involve the personal. Therefore, no ethical approval is required to use the data. We will submit the final research results to a peer-reviewed journal for publication.

## 2 Discussion

In recent years, the morbidity and mortality of CRF have increased significantly, which imposes a heavy economic burden on society.^[13,14]^ It is a long-term CRF that is associated with many factors in life, such as diet, sleep, and drug-induced injury, which can continuously cause a decline in kidney function. In terms of treatment, there is a worldwide search for effective treatments. Modern medicine advocates health education, regular diet and exercise, control of blood pressure, blood sugar and blood lipids, and anti-infection treatment for infected patients. Despite these, there are no effective drugs to lower blood creatinine and slow the progression of kidney failure. TCM has a long history and culture, which has been proven in clinical practice for thousands of years and can effectively treat CRF. The treatment method used in this study is colonic dialysis combined with TCM retention enema, so that the metabolic harmful substances produced by the body can be discharged from the intestine and the damage to the organs of the whole body can be alleviated.

Therefore, we plan to retrospectively analyze the literature on colonic dialysis combined with TCM retention enema in the treatment of CRF to find a scientific theoretical basis and provide evidence for the safety and effectiveness of clinical treatment of CRF.

## Author contributions

**Conceptualization:** Zhilei Wang, Shoulin Zhang.

**Data curation:** Xue Zheng, Lili Zhang.

**Formal analysis:** Zhilei Wang, Xue Zheng.

**Funding acquisition:** Shoulin Zhang.

**Methodology:** Zhilei Wang, Lili Zhang.

**Resources:** Shoulin Zhang.

**Software:** Zhilei Wang, Xue Zheng.

**Writing – original draft:** Zhilei Wang

**Writing – review & editing:** Shoulin Zhang.
